# Hepatitis delta virus co-infection in patients with hepatocellular carcinoma and people living with HIV in Nigeria

**DOI:** 10.1186/s12879-026-12913-7

**Published:** 2026-02-26

**Authors:** Ijeoma Ifeorah, Birgit Bremer, Yusuf Musa, Julia Kahlhöfer, Oluwatosin Oguntoye, Carey Tishiya, Gatuwa Aglavdawa, Ojonuga Ameh, Yussuf Abdulkadir, Olumuyiwa Ariyo, Manfred Anim, Andre Reinhardt, Albert Heim, Uwem George, Lukman Abdulkareem, Ibrahim Umar Garzali, Heiner Wedemeyer, Lisa Sandmann

**Affiliations:** 1 Department of Medical Laboratory Sciences, University of Nigeria, Enugu, Nigeria; 2 Center for Translation and Implementation Research, IVAN Research Institute, University of Nigeria, Enugu, Nigeria; 3 Enlightenment Initiative on Viral Hepatitis in Nigeria, Ede, Osun State Nigeria; 4 Department of Gastroenterology, Hepatology, Infectious Diseases, and Endocrinology, Hannover Medical School, Carl-Neuberg-Str. 1, 30625 Hannover, Germany; 5 Department of Internal Medicine, Federal Teaching Hospital Katsina, Katsina, Nigeria; 6 D-SOLVE consortium, an EU Horizon Europe-funded project (no. 101057917), Hannover, Germany; 7 German Center for Infection Research (DZIF), HepNet Study-House/German Liver Foundation, Hannover, Germany; 8 Department of Medicine, Afe Babalola University, Ado-Ekiti, Ekiti State Nigeria; 9 Department of Medicine, Federal Teaching Hospital Ido-Ekiti, Ido-Ekiti, Ekiti State Nigeria; 10 Urban Health Initiative, University of Chicago Medicine, Chicago, USA; 11 Department of Hematology, Federal Teaching Hospital, Gombe, Nigeria; 12 Department of Internal Medicine, University of Abuja Teaching Hospital, Abuja, Nigeria; 13 Department of Internal Medicine, Aminu Kano Teaching Hospital, Kano, Nigeria; 14 Roboscreen GmbH, Leipzig, Germany; 15 Institute of Virology, Hannover Medical School, Hannover, Germany; 16 Institute of Genomics and Global Health, Redeemer’s University, Ede, Osun State Nigeria; 17 Department of Surgery, Aminu Kano Teaching Hospital, Kano, Nigeria; 18 Excellence Cluster RESIST, Hannover Medical School, Hannover, Germany

**Keywords:** HDV, Nigeria, Hepatocellular Carcinoma, HIV/HBV co-infected, High-risk population, Epidemiology

## Abstract

**Introduction:**

Despite Nigeria’s high burden of hepatitis B virus (HBV), the epidemiological landscape of hepatitis D virus (HDV) remains largely unexplored. Consequently, HDV remains under-recognized as a public health concern, slowing down efforts to improve surveillance and clinical care. We investigated HDV infection among distinct HBV-positive populations in Nigeria, including individuals with chronic HBV infection living with or without HIV and those with hepatocellular carcinoma (HCC) and/or cirrhosis.

**Method:**

A cross-sectional study that involved 390 HBV-infected adults recruited from five tertiary-level hospitals in Nigeria. HDV total antibodies (anti-HDV) were determined in 379 samples using an automated chemiluminescent immunoassay. HDV-RNA quantification was performed in all 379 patients. Samples with detectable HDV-RNA were subjected to Sanger sequencing, and results analyzed using bioinformatics tools.

**Results:**

The overall anti-HDV antibody (HDV-Ab) seroprevalence was 8.1% with a significant difference observed across individual clinical groups: 4.1%, 11.0%, and 15.2% in asymptomatic HBV carriers, HCC/cirrhosis, HIV/HBV co-infection, respectively. Compared with asymptomatic HBV participants, HCC/cirrhosis (adjusted OR 2.71, 95% CI 1.04–7.48) and HIV/HBV (adjusted OR 3.28, 95% CI 1.20–9.25) groups had higher odds of anti-HDV positivity. HDV-RNA was detectable in 22.6% of all anti-HDV positive samples, with viral load ranging from 26.9 IU/mL to 234,000 IU/mL. HDV genotypes 1 and 5 were identified in HDV-RNA detectable samples.

**Conclusion:**

This study reveals higher anti-HDV rates in HIV/HBV coinfected individuals and suggests HDV as a significant contributor to liver cancer burden in Nigeria. These findings underscore the need for routine HDV screening among high-risk HBV-infected populations to enhance early detection and guide clinical management.

## Introduction

Globally, hepatitis D virus (HDV) accounts for an estimated one in five cases of hepatitis B virus (HBV)-associated liver disease and hepatocellular carcinoma (HCC) [1]. HDV is responsible for the most severe forms of viral hepatitis, even when it is the smallest known human viral pathogen. HDV can only occur in individuals with HBV infection because HDV requires the envelope proteins of HBV (HBsAg) for assembly and propagation [2]. HDV infection can occur either as (a) HBV/HDV coinfection, where there is a concomitant acute HBV and HDV infection followed by clearance of both viruses in about 95% of persons, or (b) superinfection in an already chronically HBV-infected person resulting in chronic hepatitis delta infection in 90% of the cases [3]. HDV super-infection of chronic HBV (CHB) accelerates the progression of HBV-related liver disease, leading to fibrosis, cirrhosis, and HCC. Discovered nearly five decades ago [4], with evolving advances in diagnostic and therapeutic landscapes, yet HDV remains a neglected research and clinical problem in Nigeria [5–7]. Limited public awareness and low clinical and research interest obscure its true burden, hindering a comprehensive understanding of the HDV impact and delaying meaningful efforts to strengthen surveillance and clinical management in the country.

The prevalence of total antibodies against HDV (anti-HDV) among HBV-infected individuals in the general population is estimated at 4.5%, representing 9–19 million infected people worldwide [2]. As expected, higher estimates are observed among patients attending hepatology clinics, with prevalence rates reported up to 16.4% [2]. Nigeria is hyperendemic for HBV, with regional estimates ranging from 5.9% to 12.9% and a reported national average of 8.1% among adults [8, 9]. This substantial HBV burden translates into a large population at risk for HDV infection. Notably, epidemiological data on HDV in Nigeria remain limited and inconsistent across regions and subpopulations. A recent nationwide study of 1,281 asymptomatic CHB patients in Nigeria reported a pooled anti-HDV prevalence of 4.6%, with regional variation ranging from 0.8% in the South-East to 9.5% in the North-East [10]. Similarly, a systematic review of studies from Nigeria (2009–2024) found anti-HDV prevalence ranging from 2.0% to 31.6% across different HBV-infected populations, including individuals co-infected with HIV, HCV, or malaria, as well as the general population [11]. Studies focusing on patients with cirrhosis and/or HCC have generally reported varying prevalences, typically between 0% and 16.4% [12, 13]. HDV is recognized for its significant genetic and evolutionary diversity, having been categorized into eight distinct genotypes (HDV-1 through HDV-8), with multiple sub-genotypes also identified [14, 15]. Although HDV-1 is the most widely distributed strain globally, other variants exhibit more limited geographical distributions: HDV-2 and HDV-4 are mainly located in Eastern and Northern Asia; HDV-3 is primarily present in South America; while HDV-5 through HDV-8 are found in Sub-Saharan Africa. In Nigeria, a few molecular studies have reported the circulation of HDV-1, HDV-5, and HDV-6 [14, 16]. 

For diagnosis, the World Health Organization (WHO) recommends an initial anti-HDV screening in all HBsAg-positive individuals, with confirmatory testing in anti-HDV–positive cases using nucleic acid amplification methods to detect active (viremic) HDV-RNA infection [17]. One of the main obstacles to HDV diagnosis in Nigeria is the lack of routine testing in healthcare facilities [7], which leaves many individuals unaware of their infection status. Limited HDV testing is not unique to Nigeria, as low rates of anti-HDV testing are reported globally, including in high-income countries [2, 6, 17]. The limited research attention devoted to HDV, coupled with weak surveillance systems, has resulted in a lack of reliable data to define the true burden of HDV infection in Nigeria, even among high-risk groups. Consequently, HDV remains under-recognized as a significant clinical problem, delaying efforts to strengthen surveillance and improve patient care. To address these gaps, we investigated HDV infection across diverse HBV-positive populations in Nigeria, including individuals with HCC, HIV coinfection, and asymptomatic chronic carriers.

## Methods

### Study design and setting

**Fig. 1:**
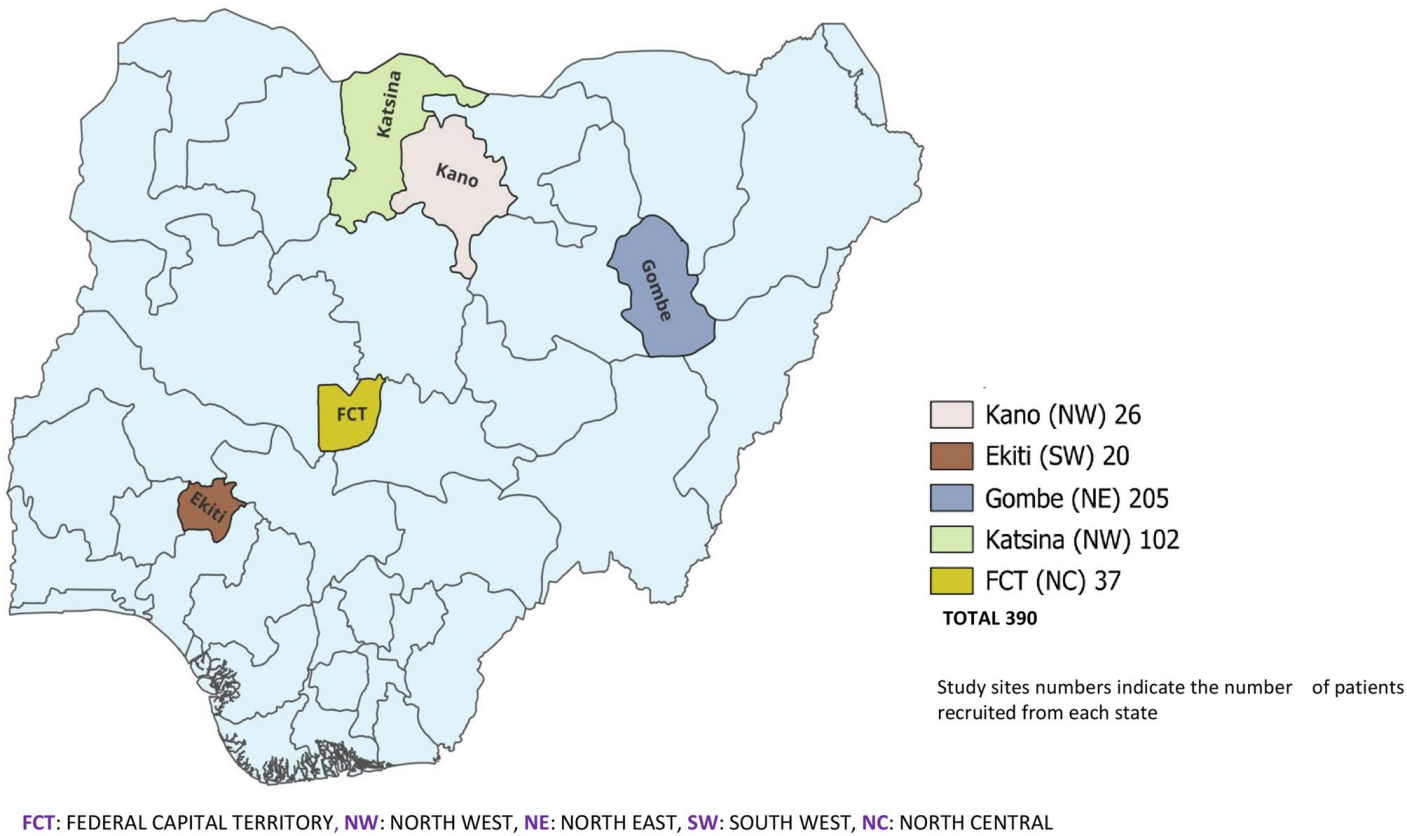
Map of Nigeria showing the study sites

We conducted a cross-sectional study between June 2021 and March 2023 among different subgroups of HBV-infected adults recruited from five tertiary-level hospitals in Nigeria (Fig. 1). The hospitals included: University of Abuja Teaching Hospital, Gwagwalada, Federal Teaching Hospital, Katsina, Federal Teaching Hospital, Gombe, Federal Teaching Hospital, Ido-Ekiti, and Aminu Kano Teaching Hospital, Kano.

### Participants and specimens

A total of 390 consenting HBsAg-positive participants were enrolled across all study sites. These comprised 124 patients with advanced liver disease (HBV-related HCC [*n* = 123] or cirrhosis without HCC [*n* = 1] confirmed by clinical evaluation and/or radiological imaging), 200 patients with asymptomatic CHB without evidence of cirrhosis or HCC, and 66 persons living with HIV (PLHIV) and HBV-coinfection with no clinical manifestations of liver disease. Except for PLHIV who were recruited from the HIV clinic, all others were recruited from the gastroenterology specialist clinics of participating centers. Whole blood samples were collected from participants into EDTA tubes, and plasma was separated and stored in duplicate aliquots at − 80 °C before shipment to Hannover Medical School (MHH), Germany, where testing was done. Demographic (age and sex) and clinical data (antiviral treatment history and duration of HIV infection) were extracted from patient medical records where available.

### Serological testing, HDV-RNA quantification and genotyping

**Fig. 2:**
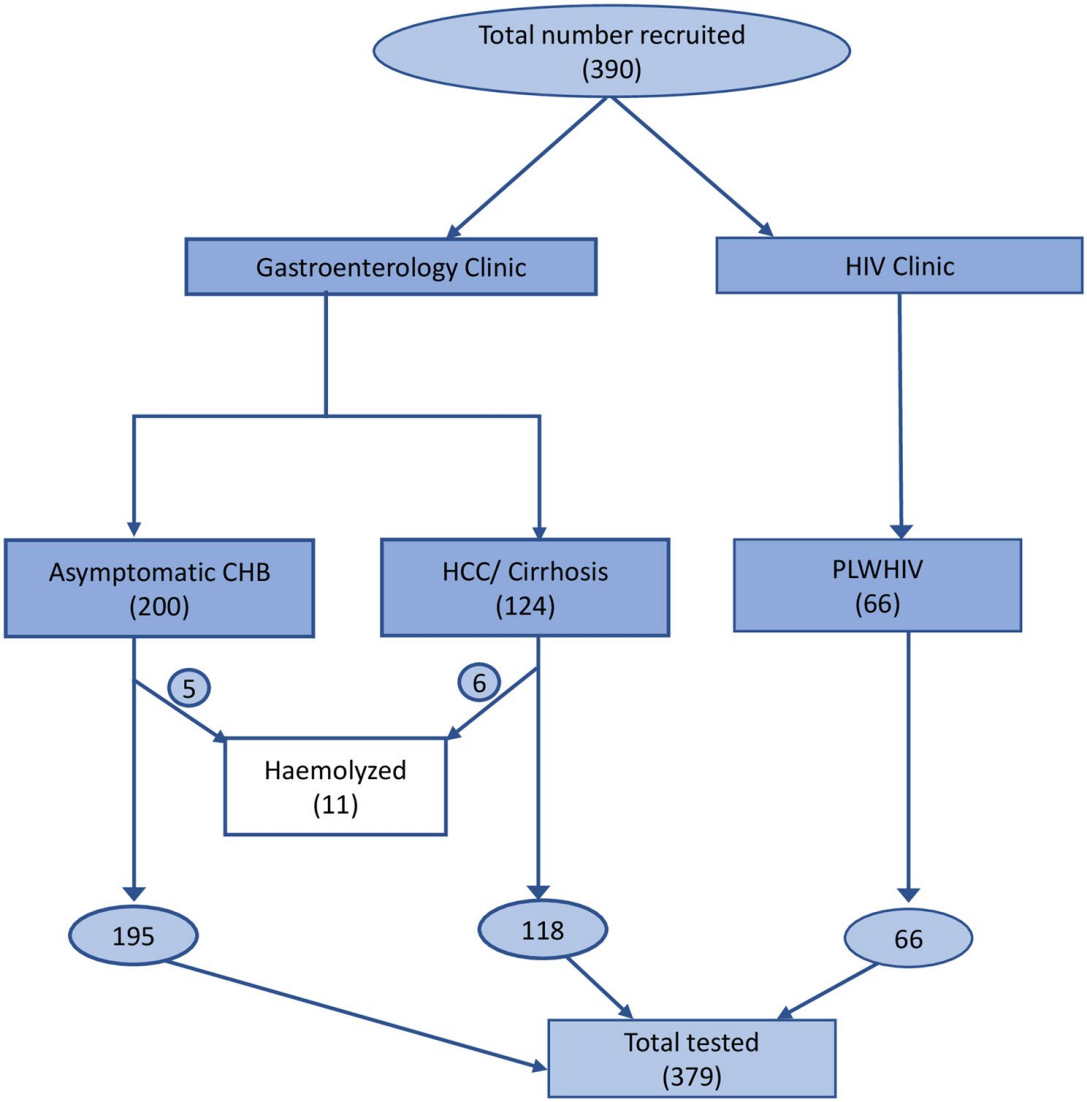
Flow diagram indicating the number of samples tested for anti-HDV and HDV-RNA

Rapid HBsAg testing (CTK Biotek, USA) and HIV screening (Determine™ HIV-1/2, Abbott) were performed on all collected samples to reconfirm HBV and HIV status. Serological testing for anti-HDV was performed using an automated chemiluminescent immunoassay (CLIA) (DiaSorin, Italy). HDV-RNA was extracted with the Roboscreen INSTANT Virus RNA/DNA Kit and quantified by real-time RT-PCR using the RoboGene HDV-RNA Quantification Kit 2.0 (Roboscreen GmbH, Germany). Due to marked hemolysis in eleven samples, testing was done in 379 patient samples (Fig. 2).

For patient samples with initial discordant results (anti-HDV negative but HDV-RNA positive), stored duplicate aliquots and, where available, second blood draws were retrieved. Repeat serological testing was then performed, and HDV-RNA quantification was repeated using both the RoboGene assay and an additional method (AltorStar 1.5). All assays were performed according to the manufacturer’s protocols.

HDV genotyping was performed on samples with detectable HDV-RNA using direct sequencing (Sanger method) of amplicons spanning the R0 region of the genome. Subsequently, sequences were compared against those in the NCBI GenBank database utilizing nucleotide BLAST (https://blast.ncbi.nlm.nih.gov/Blast.cgi, accessed on 27 August 2025) to confirm HDV genotypes. Reference sequences for all HDV genotypes (HDV-1 to HDV-8) were retrieved from the NCBI Virus database for phylogenetic analysis, which included the top five hits from the BLASTn search corresponding to each of the sequences from this study. After deduplication, the sequences were aligned using MAFFT version 7 [18] and phylogenetic analysis was performed using IQ-TREE 2 (1000 bootstrap replicates) [19] with Model Finder [20] and the resulting tree was visualized using Interactive Tree of Life (iTOL) v6 [21]. 

### Statistical analysis

Analyses were performed in R (version 4.4.2) using RStudio (version 2025.09.0). Continuous variables are summarized as medians with interquartile ranges (IQRs) and categorical variables as counts and percentages. For continuous outcomes, between-group comparisons used the Wilcoxon rank-sum test (two groups) or the Kruskal–Wallis test (three groups). For categorical outcomes, we used Pearson’s chi-square test or Fisher’s exact test when expected cell counts were < 5. Proportions (anti-HDV reactivity, HDV-RNA positivity) are reported with 95% Clopper–Pearson exact confidence intervals. To control for the potential influence of age, a sensitivity analysis restricted to participants older than 40 years was conducted, and anti-HDV prevalence was compared across clinical groups. In addition, multivariable logistic regression analysis adjusting for age and sex was performed to evaluate whether age could account for the observed differences in anti-HDV positivity across clinical groups. All tests were two-sided with an α-level of 0.05. Analyses excluded missing values from denominators where applicable.

## Results

Among 183 asymptomatic CHB cases with documented treatment records, only 41 (21.7%) individuals received anti-HBV therapy (Entecavir or Tenofovir disoproxil fumarate (TDF). None of the patients with advanced liver disease had any history of HBV treatment at the time of initial diagnosis and nearly all (122/124, 98.4%) HCC patients presented with advanced HCC (Barcelona Clinic Liver Cancer [BCLC] stage C and D). Thirty-nine (31.9%) of HCC patients who could afford out-of-pocket treatment, were offered systemic treatment (sorafenib) and antiviral treatment (TDF) while the sole cirrhotic patient was offered TDF. All 66 (100%) PLHIV were on antiretroviral treatment for both HIV and HBV. The median age of the study participants was 42 years (IQR 16) with a female to male ratio of 1:1.8. The socio-demographic characteristics and number of patients drawn from each participating site are summarized in Table 1.

**Table 1:** Demographic characteristics of study participants

Characteristics	Total *N* = 390 (%)	Asymptomatic CHB *N* = 200 (%)	HCC/cirrhosis *N* = 124 (%)	HBV/HIV (PLHIV) *N* = 66 (%)
**Age (years**,** IQR)**	42 (16)	39 (18)	48 (15)	46 (10)
**Age group (years)**				
≤40	162 (41.5)	107 (53.5)	36 (29.1)	19 (28.7)
> 40	228 (58.5)	93 (46.5)	88 (70.9)	47 (71.3)
**Sex**				
Female	141(36.2)	75 (37.5)	31 (25)	35 (53.0)
Male	249 (63.8)	125 (62.5)	93 (75)	31 (47.0)
**Number recruited from each center**				
Ekiti	21 (5)	--	21 (17)	--
FCT	46(12)	23 (12)	23 (19)	--
Gombe	194 (50)	128 (64)	--	66 (100)
Kano	27 (7)	14 (7)	13 (10)	--
Katsina	102 (26)	35 (18)	67 (54)	--
**Duration of HIV diagnosis** **(years)**				
*≤* 10	-	--	--	23 (34.8)
*≥* 10	-	--	--	43 (65.2)
**HBV treatment history**				
No Treatment		142 (77.6)*	#	0 (0 )
TDF/ETV		41 (22.4)*	#	-
DTG/TDF/3TC		-	#	66 (100)

*= Treatment record only in 183 of the Asymptomatic CHB, #= Palliative treatment, and in 39 patients who could afford out-of-pocket payment, TDF and Sorafenib were offered. Abbreviations: 3TC, Lamivudine; HCC, hepatocellular carcinoma; CHB, chronic HBV; DTG, Dolutegravir; ETV, Entecavir; FCT, Federal Capital Territory; FTC, Emtricitabine; HCC, hepatocellular carcinoma; PLHIV, persons living with HIV; TDF, Tenofovir

The overall prevalence of anti-HDV was 8.1% with significant differences across clinical groups: 4.1% in asymptomatic CHB, 11.0% in patients with HCC and/or cirrhosis, and 15.2% in the HIV/HBV co-infected cohort (*p* = 0.006). Table 2. A higher anti-HDV prevalence of 11.0% was observed in patients aged > 40 years in comparison with 4.4% seen in patients aged *≤* 40 years (*p* = 0.034). Subgroup analysis revealed a significant association between older age (> 40 years) and anti-HDV positivity in asymptomatic CHB, with rates of 7.9% and 0.9% in > 40 and *≤* 40 years, respectively (*p* = 0.025). No significant differences in anti-HDV prevalence were detected in the HCC/cirrhosis and PLHIV clinical subgroups when stratifying according to age (Table 2). Overall, numerically higher anti-HDV prevalence was detected in females (11.6%) compared to male (6.2%) participants (*p* = 0.101). This trend was present in all clinical subgroups (Table 2).

**Table 2:** Summary of HDV seroprevalence in the study population

Study Site	Total			Asymptomatic CHB			HCC/cirrhosis			HBV/HIV (PLHIV)			
	Enrolled	Tested	Anti-HDV (%)	Enrolled	Tested	Anti-HDV (%)	Enrolled	Tested	Anti-HDV (%)	Enrolled	Tested	Anti- HDV (%)	
Ekiti (SW)	20	20	3 (15.0)	--	--	--	20	20	3 (14.3)	--	--	--	
FCT (NC)	37	31	4 (12.9)	13	10	1 (5.0%)	24	21	3 (15.0)	--	--	--	
Gombe (NE)	205	203	17 (8.4)	139	137	7 (5.5%)	--	--	--	66	66	10	
Kano (NW)	26	25	0	14	13	0	13	12	0 (0)	--	--	--	
Katsina (NW)	102	100	7 (8.4)	35	35	0	67	65	7 (10.8)	--	--	--	
**Total**	390	379	31 (8.1)	200	195	8 (4.1%)	124	118	13 (11.0)	66	66	10 (15.2)	*p*= 0.006
**Sex**													
Male	249	241	15 (6.2)	125	120	4 (3.3%)	93	90	8 (8.9)	31	31	3 (9.7)	
Female	141	138	16 (11.6)	75	75	4 (5.3%)	31	28	5 (17.9)	35	35	7 (20.0)	
			*p* = 0.101			*p* = 0.487			*p* = 0.299			*p* = 0.314	
**Age (Years)**													
*≤* 40	162	160	7 (4.4)	107	106	1 (0.9)	36	35	4 (11.4)	19	19	2 (10.5)	
> 40	228	219	24 (11.0)	93	89	7 (7.9)	88	83	9 (10.8)	47	47	8 (17.0)	
			*p* = 0.034			*p* = 0.025			*p* = 1.000			*p* = 0.711	
**Median age (years**,** IQR)**													
Anti-HDV+	48 (11)			48 (15.3)			48 (22)			48 (4.5)			
Anti-HDV-	42 (16)			39 (17)			47 (14)			46 (10.3)			

Abbreviations: HCC, hepatocellular carcinoma; CHB, chronic HBV; IQR, Interquartile range; NC, North-Central; NE, North-East; NW, North-West; PLHIV, persons living with HIV; SW, south-west

Because anti-HDV seropositivity was associated with older age in unadjusted analyses and age distributions differed across diagnostic groups, we conducted age-restricted and age-adjusted sensitivity analyses. When analyses were restricted to participants older than 40 years, differences in anti-HDV prevalence across clinical groups were attenuated and no longer statistically significant (*p* = 0.27). In multivariable logistic regression, adjusting for age and sex, the clinical group remained independently associated with anti-HDV seropositivity. Compared with individuals with asymptomatic chronic HBV infection, higher odds of anti-HDV positivity were observed among participants with HCC/cirrhosis (adjusted OR 2.71, 95% CI 1.04–7.48) and those with HIV/HBV coinfection (adjusted OR 3.28, 95% CI 1.20–9.25). Age was not independently associated with anti-HDV positivity in adjusted models.

HDV-RNA was detectable in 22.6% (7/31) of anti-HDV-positive individuals across all clinical subgroups, with viral loads ranging from 26.9 IU/mL to 234,000 IU/mL. Significantly lower rates of HDV viremia were observed for the asymptomatic CHB (1/8, 12.5%) and HCC/cirrhosis subgroups (1/13, 7.7%) compared to the much higher rate of 50% (5/10) in the subgroup of PLHIV (*p* = 0.0405). Notably, in seven cases with detectable HDV-RNA but negative HDV antibody testing on initial test, repeated testing confirmed the absence of both anti-HDV and HDV-RNA in all samples (Table 2). Furthermore, we observed that the median duration of HIV infection was modestly longer in anti-HDV-positive compared to anti-HDV-negative patients (15 years [IOR 6.5] vs. 11.5 years [IQR 5.0], *p* = 0.301). A similar trend was observed for HDV-RNA-positive compared to HDV-RNA-negative PLHIV (14 years [IQR 6.5] vs. 12 years [5.5], *p* = 0.295) (Table 3).

**Table 3:** Distribution of HDV RNA, genotype, and association of HDV infection markers with HIV duration in the study population

HDV RNA ^#^	Total		Asymptomatic CHB		HCC/cirrhosis		HBV/HIV (PLHIV)		
	Tested (*n*)	HDV RNA + (*n*, %)	Tested (*n*)	HDV RNA + (*n*, %)	Tested (*n*)	HDV RNA + (*n*, %)	Tested (*n*)	HDV RNA + (*n*, %)	
	31	7 (22.6)	8	1 (12.5)	13	1 (7.7)	10	5 (50)	*p* = 0.0405
HDV viral load	26.9–234,000 (IU/mL)		32 (IU/mL)		234,000 (IU/mL)		26.9–3610 (IU/mL)		
HDV genotype	HDV-1 (83.3%), HDV-5 (16.6%)		HDV-5		HDV-1		HDV-1		
						Duration of HIV infection	Years (Median, IQR)		
						Anti-HDV+	11.5 (5.0)		*p* = 0.301
						Anti-HDV -	15.0 (6.5)		
						HDV RNA +	14.0 (6.5)		*p* = 0.295
						HDV RNA -	12.0 (5.5)		

# = Wilcoxon rank sum test

Abbreviations: HCC, hepatocellular carcinoma; CHB, chronic HBV; PLHIV, persons living with HIV

**Fig. 3:**
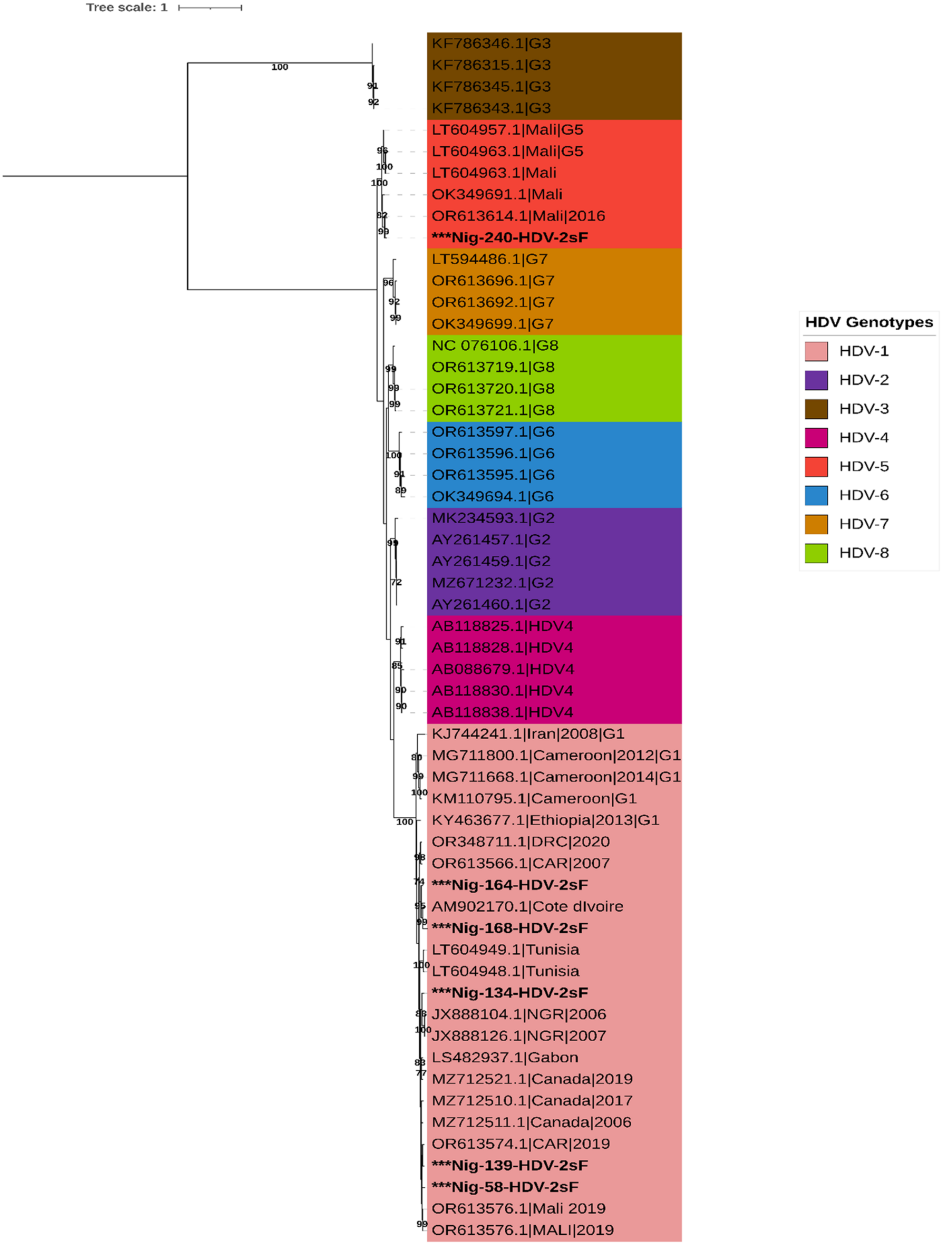
A maximum likelihood phylogenetic tree inference for sequences detected in this study and reference sequences across 8 genotypes for HDV. All the HDV genotypes are colour-coded as shown in the legend, and sequences from this study are in bold black colour with an asterisk

HDV sequences were obtained in six of the seven samples with detectable HDV-RNA (Table 3). Phylogenetic analysis revealed the circulation of HDV genotype 1 (*n* = 5) and genotype 5 (*n* = 1) (Fig. 3). The HDV-5 sequences from this study clustered with previously described sequences from Mali, while the HDV-1 sequences from this study formed 3 subclusters with previously reported HDV-1 sequences from countries in Africa, including Nigeria, Tunisia, Mali, the Central African Republic, and Côte d’Ivoire.

## Discussion

This large screening of both asymptomatic CHB patients and distinct cohorts at risk revealed an overall HDV-Ab prevalence of 8.1% with significant variation in HDV-Ab and HDV-RNA distribution across clinical subgroups. Our findings suggest that Nigeria is a country of low HDV prevalence, but with higher rates in individuals with HCC and HIV coinfection. This study provides new insights into the epidemiology of HDV among homogenous subgroups of HBV-infected populations and highlights that HDV, which has long been overlooked, is a significant contributor to HCC and an important clinical concern among PLHIV in Nigeria.

The low anti-HDV prevalence of 4.1% obtained in the study among the asymptomatic CHB subgroup is similar to prevalences ranging from 1.1% to 5.7% reported in similar cohorts from studies conducted within the last decade from different regions in Nigeria [22–27]. Conversely, higher anti-HDV prevalences of 10.8% and 31.6% have also been reported in similar cohorts by others [28, 29]. HDV coinfection of CHB is known to accelerate disease progression, leading to cirrhosis and HCC. Therefore, HDV-infected people are more likely to present with advanced disease than non-HDV-infected ones, which may explain the higher prevalence observed in HCC/cirrhosis than asymptomatic CHB. In addition, PLHIV are regarded as a high-risk group for HDV infection and HCC [2, 30]. The 11% prevalence seen in the HCC/cirrhosis group, though lower than the pooled 16.4% reported by Stockdale et al. [2], is comparable to the prevalence presented in a review including few studies from the WHO African region (12.3% [95% CI: 10.1–14.7]), but higher than the data from Nigeria presented in the same review (4.7% [95% CI: 3.2–7.8]) [2]. An earlier multicenter study conducted in several countries in Sub-Saharan Africa reported anti-HDV prevalence rates of 1.3% and 6.7% from liver disease patients from southwestern Nigeria [31]. The 15.1% anti-HDV prevalence observed among PLHIV in this study compares to 10.8% reported in the north-western part of the country [28]. Lower rates of 5.6% and 6.7% have been reported from other parts of the country [24, 32]. The observed differences in reported prevalence between cohorts from this study and previous studies may be due to differences in sample size, notable regional variations in HDV prevalence, as well as assay variation. Most of the HCC samples were collected from northern Nigeria, while the previous reports were mainly from southwestern Nigeria. Furthermore, HDV antibody assays have evolved over the years, and possible variation in assay performance between earlier studies is expected. The higher anti-HDV prevalence from HCC/cirrhosis and PLHIV subgroups, with higher adjusted odds of anti-HDV positivity observed in these subgroups compared with those with asymptomatic chronic HBV infection in this study, suggests that HDV contributions to liver disease progression in CHB may have been overlooked hitherto and underscores the need for HDV surveillance in CHB and PLHIV. Significantly higher anti-HDV prevalence was observed in the older age group (> 40 years) in the entire study population and the asymptomatic CHB subgroup in particular (Table 2). This is consistent with findings from other studies in Nigeria [28] and across the world [33, 34] with reported higher prevalence rates in older individuals. Specifically, a prospective study conducted in Iran reported a yearly increase of 6% for anti-HDV positive individuals in a cohort of HBV positive individuals [33]. However, some studies in Nigeria showed higher anti-HDV prevalence in those younger than 40 years [25, 29], while others have shown no difference [22, 23, 35]. With regard to sex, numerically higher anti-HDV rates were observed among females compared to males across all subgroups. Studies from Nigeria and other parts of the world have also documented higher anti-HDV prevalence rates in females than males [34, 35]. In contrast, few studies have reported higher anti-HDV prevalence in males compared to females [25, 36]. 

Low rates of HDV-RNA viremia were noted, as only 22.6% of all anti-HDV positives had detectable RNA despite the absence of HDV-directed antiviral treatment. This low rate of HDV-RNA positivity among the Sub-Saharan African population is well established [10, 24, 35, 37]. But factors that could be responsible for the limited viral replication are not well understood [10]. Interestingly, 50% of PLHIV in this study were viremic, supporting existing evidence from Nigeria and other Sub-Saharan African countries that HIV/HBV co-infected individuals are more likely to have active HDV infection compared to HIV negative counterparts [11, 24, 37]. HIV related immune dysregulation may partly explain this observation. Although the duration of HIV infection may not be directly associated with HDV risk, long-standing HIV infection may pose longer cumulative exposure to risk factors for the acquisition of other co-morbidities, including HDV in PLHIV. The absence of an observed association between the duration of HIV and HDV infection status may be explained by the fact that PLHIV who participated in this study were recruited from a facility providing routine HIV care, including treatment and monitoring. We can therefore assume a well-controlled HIV infection in the participants. Nonetheless, we are limited by the size of the PLHIV cohort in the present study; a larger prospective study involving a similar cohort would be beneficial to elucidate factors involved in the HIV/HBV/HDV disease conundrum. In addition, a wide margin between RNA titers was observed across the subgroups, with the highest titers, 234,000 IU/mL, in the HCC/cirrhosis subgroup. It is important to note that HDV viral titers do not often reflect disease severity, as HDV can drive advanced liver pathology even at low or fluctuating RNA levels [38]. 

The discordant result (anti-HDV^_^ /HDV-RNA+) observed in our initial test is not an isolated occurrence, as Opaleye et al. [24] have reported cases of HDV-RNA positivity despite undetectable anti-HDV in Nigeria. Furthermore, discussions on the possibility of the existence of HDV genotypes (especially of African origin) that are not detected by the currently available testing platforms have been ongoing among the HDV research community it [39]. Using stored replicate sample vials or a second blood draw, the presence of HDV-RNA-positive/anti-HDV-negative samples could not be confirmed. Thus, human error at any stage from sample preparation to final RNA quantification may have contributed to the initial discordant test outcomes. Similarly, in a separate study that examined the possible presence of detectable HDV-RNA in anti-HDV-negative samples using 914 archived samples from Senegal. No HDV-RNA-positive/anti-HDV-negative cases were reported [40]. Our findings support current algorithms that limit HDV-RNA testing to anti-HDV-positive individuals. The detection of HDV genotype 1 and 5 is consistent with the genotype distribution reported in West Africa [37]. We noted the presence of various genotype 1 lineages, which have been documented as the most prevalent and genetically diverse among the eight HDV genotypes [16], implying the continuous circulation and evolution of HDV in Nigeria.

Our study has some limitations. First, the modest sample size, particularly of PLHIV, may have reduced the statistical power of the study. Secondly, as the study was conducted in selected centers, some centers had a small number of participants; thus, the study findings may not fully represent the national HDV picture, especially in the un/under-sampled regions. Lastly, because the study design was cross-sectional, we could not assess the temporal dynamics of HDV infection and its impact on disease progression. Despite these limitations, the major strength of the study is in the homogenous nature of the study cohorts that enabled reliable estimation of HDV prevalence across distinct clinical subgroups. Also, the ability to exclude occult HDV infection using stored replicates and/or freshly drawn plasma strengthened the validity of our conclusion.

## Conclusions

In summary, anti-HDV seroprevalence differed across clinical groups, with higher adjusted odds observed among individuals with HCC/cirrhosis and HIV/HBV coinfection compared with those with asymptomatic chronic HBV infection. Although age distributions differed across groups and attenuated unadjusted comparisons in restricted analyses, group-level differences persisted after multivariable adjustment. However, these findings should be interpreted cautiously given sample size limitations and baseline heterogeneity. Still, the findings underscore the urgent need to incorporate HDV screening into HBV management, especially among high-risk groups in Nigeria. Uncertainties remain with respect to estimating the true national HDV burden, temporal dynamics of infection, and determinants of disease progression in Nigeria and similar settings. Future large-scale and longitudinal studies using representative cohorts are needed to fill these gaps. Lastly, incorporating HDV screening into routine HBV and HIV care is critical to identify high-risk patients for targeted interventions.

## Data Availability

The HDV RNA sequences generated for HDV genotyping of the current study are available at GenBank with the following accession numbers: PX620868;PX620869; PX620870; PX620871;PX620872;PX620873. Other data and analyses of the current study are available from the corresponding author on reasonable request.

## Abbreviations

Anti-HDV: Antibodies to Hepatitis D Virus
CHB: Chronic Hepatitis B
CLIA: chemiluminescent immunoassay
FCT: Federal Capital Territory
HBV: Hepatitis B Virus
HCC: Hepatocellular Carcinoma
HDV: Hepatitis D Virus
HDV-RNA: Hepatitis Virus Ribonucleic Acid (Genome)
HIV: Human Immunodeficiency Virus
NCBI: National Center for Biotechnology Information
WHO: World Health Organization
